# Osteoporosis, inflammation and ageing

**DOI:** 10.1186/1742-4933-2-14

**Published:** 2005-11-04

**Authors:** Lia Ginaldi, Maria Cristina Di Benedetto, Massimo De Martinis

**Affiliations:** 1 Department of Internal Medicine, University of L'Aquila, Italy

**Keywords:** Ageing, Inflammation, Cytokines, Bone remodelling, Osteoporosis

## Abstract

Osteoporosis is a condition characterized by low bone mass and increased bone fragility, putting patients at risk of fractures, which are major causes of morbidity substantially in older people. Osteoporosis is currently attributed to various endocrine, metabolic and mechanical factors. However, emerging clinical and molecular evidence suggests that inflammation also exerts significant influence on bone turnover, inducing osteoporosis. Numerous proinflammatory cytokines have been implicated in the regulation of osteoblasts and osteoclasts, and a shift towards an activated immune profile has been hypothesized as important risk factor. Chronic inflammation and the immune system remodelling characteristic of ageing, as well as of other pathological conditions commonly associated with osteoporosis, may be determinant pathogenetic factors. The present article will review the current perspectives on the interaction between bone and immune system in the elderly, providing an interpretation of osteoporosis in the light of inflamm-ageing.

## Introduction

Osteoporosis is a relevant age related disorder, characterized by low bone mass and increased bone fragility putting the patient at risk for fractures [1]. Currently osteoporosis is viewed as a heterogeneous condition which can occur in any age of life and its etiology is attributed to various endocrine, metabolic and mechanical factors (abnormalities of parathyroid hormone and calcitonin secretion, insufficient vitamin D and calcium intake, postmenopausal hormonal condition, pregnancy, nutritional disorders, immobility and consumption of drugs such as cortisone, among others) [2]. Recently, growing understanding of the bone remodelling process suggests that factors involved in inflammation are linked with those critical for bone physiology and remodelling, supporting the theory that inflammation significantly contributes to the aetiopathogenesis of osteoporosis [3,4].

## Is osteoporosis an inflammatory process?

Clinical observations reveal coincidence of systemic osteoporosis with period of systemic inflammation as well as co-localization of regional osteoporosis with areas of regional inflammation [2]. Different epidemiologic studies report an increase in the risk of developing osteoporosis in various inflammatory conditions [5-8]. Immunological dysfunctions, autoimmune and chronic inflammatory diseases [9], HIV infection [10], hyper-IgE syndrome [11], rheumatoid arthritis [12], haematological diseases, particularly myeloma [13], and inflammatory bowel diseases [14], are associated with osteoporosis.

Erosions seen in conditions such as gout, osteomyelitis, rheumatoid arthritis, ankylosing spondylitis, and psoriatic arthritis, are typically associated with inflammation in the joints. Pro-osteoclastic cytokines, such as tumour necrosis factor (TNF)-α and interleukin (IL)-6, are elevated in these conditions and local cytokine profile is consistent with the cytokines that modulate bone resorption [15,16].

C-reactive protein (CRP) production in the liver is upregulated by IL-1, IL-6 and TNF-α, and is regarded as a sensitive marker of systemic inflammation [17,18]. An association between circulating high sensitive (hs)CRP level and bone mineral density has been observed in several immune and inflammatory diseases, as well as in healthy individuals, suggesting a relationship between subclinical systemic inflammation and osteoporosis [19].

Rheumatoid arthritis (RA) is a typical example of the link between inflammation and osteoporosis. Bone loss in RA occurs both in the joints and throughout the skeleton as a result of the release of proteinases (metalloproteinases) and proinflammatory cytokines (IL-1, TNF-α), which are responsible for cartilage and bone destruction. As a result, disease activity is an independent risk factor for osteoporosis in RA [20].

A temporal link between inflammation and osteoporosis also emerges in conditions such as ageing, menopause, pregnancy, transplantation and steroid administration. While nutritional, mechanical and hormonal factors clearly play a role in many of these situations, the concordance of osteoporosis and inflammation is buttressed by emerging molecular evidence of mediating immunological factors [2].

On the other hand, one intriguing aspect of immunosenescence is the increased production of pro-inflammatory cytokines with age and a close link between age-related systemic inflammatory process (inflamm-ageing)[21] and osteoporosis is well documented [22].

## A molecular scenario of immune regulated bone loss

Although osteoporosis is not typically considered an immunological disorder, recent data have indicated overlapping pathways between bone biology and biology of inflammation [23-25]. Certain pro-inflammatory cytokines play potential critical roles both in the normal bone remodelling process and in the pathogenesis of perimenopausal and late-life osteoporosis [26]. For example, interleukin (IL)-6 promotes osteoclast differentiation and activation [27]. This cytokine is involved in the pathogenesis of various metabolic bone diseases, including postmenopausal osteoporosis, Paget's disease and osteoporosis associated with heamtologic malignancies [28]. IL-1 is another potent stimulator of bone resorption [29] that has been linked to the accelerated bone loss seen in idiopathic and postmenopausal osteoporosis [30]. TNF-α is implicated in tumour-induced bone resorption and non-tumour-induced osteopenia [31]. Anti-TNF drugs, currently used in the therapy of several immunological disorders, are also useful in preventing and/or reversing systemic bone loss associated to the disease, as they target both the bone and the inflammatory process [20]. The production of IL-1, IL-6, and/or TNF-α by peripheral blood monocytes has been positively correlated with bone resorption or spinal bone loss in healthy pre- and postmenopausal women [32].

The inflammatory mediator nitric oxide (NO) is also involved in the pathogenesis of osteoporosis. The activation of the inducible NO synthesis (iNOS) pathway by cytokines, such as IL-1 and TNF-α, inhibits osteoblast function in vitro and stimulates osteoblast apoptosis [33].

The TNF-family molecule RANKL and its receptor RANK (receptor activator of NFkB ligand) have been specifically implicated in the bone loss in rheumatoid arthritis [34]. They are key regulators of bone remodelling and are essential for the development and activation of osteoclasts. Intriguingly, RANKL/RANK interactions also regulate T cell/dendritic cell communication, dendritic cell survival and lymphnode formation [35]. Calciotropic factors such as vitamin D3, PGE2, IL-1, IL-11, TNF-α and glucocorticoid induce RANKL expression on osteoblasts [36]. RANKL binding to the RANK expressed on haematopoietic progenitors activates a signal transduction cascade that leads to osteoclast differentiation. Moreover, RANKL stimulates bone resorbing activity in mature osteoclasts via RANK. When RANK is activated, it sends signals into the cells through tumor necrosis factor receptor-associated factors (TRAFs), mainly TRAF6. These RANK associated molecules, through downstream pathways such as NF-kB, JNK/SAPK, p38 and Akt/PKB, regulate bone resorption, activation, survival, and differentiation of osteoclasts and dendritic cells.

Osteoprotegerin (OPG), also known as osteoclastogenesis inhibitory factor, functions as a soluble decoy receptor to RANKL and competes with RANK for RANKL binding. TGFβ released from bone during active bone resorption has been suggested as a feedback mechanism by upregulating OPG level. Estrogen can enhance OPG production on osteoblasts which is a possible explanation of postmenopausal osteoporosis following estrogen withdrawal [35]. Since the expression of RANKL/RANK can be controlled by sex hormones, it is possible to speculate that this system may control gender specific differences in immunity and could be involved in the higher incidence of autoimmune diseases and osteoporosis in women. RANK-L, RANK and OPG therefore configure interesting molecular links between bone remodelling, immunity and inflammation.

## The emergence of osteoimmunology

There exists an intimate interplay between the bone and the immune system which has been termed osteoimmunology [3]. The activity of immune cells affects the balance of bone mineralization and resorption carried out by the opposing actions of osteoblasts and osteoclasts [37].

Dendritic cells, specialized to present antigens, and osteoclasts, specialized to resorb bone, share the same bone marrow precursors of the monocyte lineage and exhibit parallel lifecycles, regulated by a variety of cytokines, transcription factors and inflammatory mediators. Molecules that regulate osteoclastogenesis are key factors in many immunological functions. For example, TRAF6 functions as a molecular bridge spanning adaptive immunity, innate immunity and osteoimmunology [38]. TRAF6-deficient mice lack osteoclasts and concomitantly have defects in cytokine production and T cell stimulation [39].

Activated T cells affect bone physiology producing cytokines that lead to RANKL expression on osteoblasts. Moreover, activated T cells directly express and produce RANKL that induces osteoclast formation and activation through its specific receptor [28,35]. There are multiple mechanisms and interactions by which cytokines regulate bone resorption. IL-6 contributes to RANKL upregulation in osteoblastic cells. IL-1 and TNF-α may not only promote osteoclast generation, but they also appear to stimulate mature osteoclasts to perform more resorption cycles through modulation of RANKL activity. IL-1 is further involved in bone metabolism as an osteoblast activator: osteoblasts secrete RANKL which promotes survival and differentiation of the osteoclast precursors to mature osteoclasts through RANK. IL-1 and IL-6 also directly enhance osteoclast activity by RANKL-independent mechanisms. They may directly extend the lifespan of the osteoclasts by inhibiting osteoclast apoptosis. Both TNF-α and IL-1 inhibit collagen synthesis in osteoblasts and enhance degradation of the extracellular matrix [18]. In inflammatory or autoimmune disease states, activated T cells produce RANKL and pro-inflammatory cytokines, all of which can induce RANKL expression in osteoblasts [40]. However, the constant activity of T cells fighting the universe of antigens to which we are exposed does not usually cause extensive bone loss. Multiple T cell-derived cytokines, such as IL-12 and IL-18, might be able to interfere with RANK signalling and therefore with osteoclastogenesis and osteoclast functions. A crucial counter-regulatory mechanism whereby activated T cells can inhibit osteoclast development and activation is through the action of the antiviral cytokine interferon (IFN)-γ. Mechanistically, IFN-γ activates the ubiquitin-proteasome pathway within the osteoclasts, resulting in the degradation of TRAF6 [38].

## Ageing and osteoporosis: immunological links

Inflamm-ageing may, at least partially, be a common mechanism for the development of lower bone mass and other age-related disorders [41,42]. Senility is in fact notable for acceleration of diseases that are increasingly attributed to inflammation, such as atherosclerosis, Alzheimer's disease and asthma [21,43,44]. Many cytokines, including IL-6, TNF-α, IL-1, are elevated during senescence and play direct roles in the pathogenesis of these diseases [45]. All these cytokines are stimulators of osteoclast activity as well. Associations between atherosclerosis and osteopenia have been well documented [46,47]. These findings suggest a potential causal relationship between systemic inflammation that is observed in the elderly and the prevalence of generalized age-related osteoporosis [2]. Moreover, the increased catabolic signal, driven by inflammation also in the absence of clinically diagnosable inflammatory diseases [44], could be able to induce osteoblast apoptosis [48], as well as apoptosis of muscle cells, leading to age-related osteoporosis and sarcopenia respectively.

During ageing, under the influence of the lifelong exposure to chronic antigenic load and oxidative stress, the physiological counter-regulatory process which inhibits bone resorption following T cell activation is likely impaired. This would contribute, together with the age-related systemic low-grade inflammation, to the increasing incidence of osteoporosis during senescence.

Osteoporosis, as well as other age-related disorders, has a strong genetic component and the rate extent of bone loss during senescence varies widely between individuals. It is tempting to postulate that this may be in part due to individual differences in cytokine activity. In support of this hypothesis it has been demonstrated that certain IL-6 polymorphisms are able to influence the risk of osteoporosis in postmenopausal women [49]. Similarly, IL-1β and IL-1 receptor antagonist (IL-1Ra) gene polymorphisms are associated with reduced bone mineral density and predispose women to osteoporosis at the lumbar spine [50].

Moreover, also the sex hormonal decline which accompanies ageing contributes to the pathogenesis of senile osteoporosis through immunologically mediated mechanisms. It has been postulated that estrogens exert their effect on bone not only by direct action per se, but also by inhibiting IL-6 gene expression. A similar relationship between androgen and IL-6 gene expression also exists [51]. The decline in ovarian function is associated with decreased OPG production and spontaneous increases in proinflammatory and pro-osteoclastic cytokines such as IL-6, TNF-α, and IL-1 [32,52,53].

## Osteoporosis within an evolutionary perspective

During evolution of multi-cellular organisms, the increasing complexity of emerging systemic functions, such as inflammation, may have led to a widening range of demand for key minerals such as calcium and phosphate and storage functions may have evolved to provide mineral reservoirs [2]. Moreover, calcium is an important component of milk and its transport from mother to the fetus and neonate is a vital process to preserve species. Intriguingly, RANKL and RANK also play essential roles in the formation of a lactating mammary gland in pregnancy. This could partly contribute to both the immune remodelling and the accelerated bone loss during pregnancy and lactating [35]. Bone can thus be viewed as a mobilizable reservoir of calcium and phosphate as salts. Over time, the regulation of bone turnover was probably optimized evolutionarily to address the combined metabolic and structural demands of the host.

In this perspective osteoporosis may reflect a state of disequilibrium between structural demand for calcium and phosphate and their biologic demand during metabolically active states such as inflammation. Inflammation is the leading force driving immunosenescence and the chronic low-grade inflammatory state characteristic of ageing represents the predisposing substrate on which osteoporosis, as well as other age-related diseases, might emerge [21,54].

The lack of evolutionary selective pressure post-reproduction may have further exacerbated this disequilibrium in modern times and many biologic functions acquired during evolution have become maladaptive. Presently, in most developed countries, the human lifespan is greatly increased, and many individuals are living into post-reproductive senescence, an evolutionarily naive life epoch. Obesity, diabetes, Alzheimer's disease and atherosclerosis are examples of diseases of modernity that are attributable to modern living circumstances or that are unmasked during senility, and also the emergence of osteoporosis as a modern disease may be an example of this phenomenon [2,21,44].
